# A-MADMAN: Annotation-based microarray data meta-analysis tool

**DOI:** 10.1186/1471-2105-10-201

**Published:** 2009-06-29

**Authors:** Andrea Bisognin, Alessandro Coppe, Francesco Ferrari, Davide Risso, Chiara Romualdi, Silvio Bicciato, Stefania Bortoluzzi

**Affiliations:** 1 University of Padova, Department of Biology, Via G. Colombo 3, 35121, Padova, Italy; 2 University of Padova, Department of Statistical Sciences, via C. Battisti2 41, 35121 Padova, Italy; 3 University of Modena and Reggio Emilia, Department of Biomedical Sciences, Via G. Campi 287, 41100, Modena, Italy

## Abstract

**Background:**

Publicly available datasets of microarray gene expression signals represent an unprecedented opportunity for extracting genomic relevant information and validating biological hypotheses. However, the exploitation of this exceptionally rich mine of information is still hampered by the lack of appropriate computational tools, able to overcome the critical issues raised by meta-analysis.

**Results:**

This work presents A-MADMAN, an open source web application which allows the retrieval, annotation, organization and meta-analysis of gene expression datasets obtained from Gene Expression Omnibus. A-MADMAN addresses and resolves several open issues in the meta-analysis of gene expression data.

**Conclusion:**

A-MADMAN allows i) the batch retrieval from Gene Expression Omnibus and the local organization of raw data files and of any related meta-information, ii) the re-annotation of samples to fix incomplete, or otherwise inadequate, metadata and to create user-defined batches of data, iii) the integrative analysis of data obtained from different Affymetrix platforms through custom chip definition files and meta-normalization. Software and documentation are available on-line at .

## Background

Public databases of microarray gene expression data have been quickly growing as the use of high-throughput techniques has become routine in genome-wide studies. Major repositories of microarray data, e.g. Gene Expression Omnibus [1], ArrayExpress [2], or Stanford Microarray Database [3], are exceptionally rich mines of genomic information and exploiting their content, through meta-analysis, represents an unprecedented opportunity to improve the interpretation and validation of expression studies. Meta-analysis of large microarray expression datasets allows researchers to confirm biological hypotheses, formulated from results of a study, in a relatively inexpensive way, i.e. using data independently obtained in another laboratory, without the need of novel experiments. Meta-analysis also offers the opportunity of re-analyzing formerly available data, in combination with new samples and state-of-the-art computational methods, thus increasing the reliability and robustness of results. Finally, meta-analysis enhances the capabilities of bioinformatics methods to obtain precise estimates of gene expression differentials and to assess the heterogeneity of overall estimates.

### Challenges of integrative analysis of expression data

In recent years, different strategies to combine results from independent but related studies have been proposed. The choice of the most effective meta-analysis technique depends on the type of response (e.g., binary, continuous, survival) and on the objective of the study. Meta-analysis strategies can be divided into two broad classes: data integration and data combination. Statistical techniques as vote counting [4], p-value or rank combination [5-7] and effect size estimation [8] have been used for meta-analyses based on data integration. Instead, data combination encompasses the direct comparison of different studies, is applicable only when expression profiles have been obtained using the same array technology (e.g. Affymetrix, Agilent, Illumina, etc.) and requires an *ad-hoc *normalization step of the raw data files.

Despites numerous efforts, mining and analyzing publicly available microarray data still represents a bioinformatics challenge and the lack of appropriate tools able to overcome critical issues, as annotation, cross-platform comparison and handling of metadata, is still hampering the potentialities of large-scale meta-analyses. Performing a meta-analysis of independent microarray studies requires to carefully handle the heterogeneity of array designs, which complicates cross-platform integration, and of sample descriptions, which impact the correct characterization of specimens. At least for the case of Affymetrix arrays, cross-platform comparison has partially been solved by the adoption of custom Chip Definition Files (custom-CDF) which, relating probe sequences to annotated entities as genes or transcripts, allow matching expression profiles across subsequent generations of microarrays [9-11]. Instead, retrieval, organization and utilization of meta-information is still an extremely critical step which impacts the correct match between raw data files and sample IDs and the organization of samples into meaningful, homogeneous groups. This task is further complicated by the fact that i) datasets may be incompletely annotated, ii) the relationship between specimen, biological sample, phenotypic characteristics and raw data files, the most granular object in repositories, may be not sufficiently explicit, and iii) the procedures for managing large numbers of data files and related meta-information are tedious and error prone [12].

### Available tools

Different computational tools have been proposed for retrieval and meta-analysis of microarray expression data [13-21]. In particular, GEOSS (formerly GeneX) is a web-interfaced system for the storage and local analysis of microarray data [13]; CrossChip uses probe by probe sequence comparison to integrate data from different generations of Affymetrix arrays [14]; the server version of SeqExpress has a GEO integration tool which allows for retrieval and analysis of GEO Data Sets and GEO Series [15]; GenePattern contains a module (*GEOImporter*) for importing single GEO Data Sets or Series [16]; WGAS is a multi-purpose web-based analysis system [17]; GEOquery is a Bioconductor package for downloading and parsing SOFT files from GEO [18]; EzArray, is an Affymetrix-centered analysis system [19]; Microarray Retriever (MaRe) allows batch retrieval of microarray data from GEO and ArrayExpress according to user-defined searching criteria [20]; EMAAS allows importing and analyzing data from several repositories including GEO [21]. Some of these tools provide a user friendly interface for searching and retrieval of data from GEO and ArrayExpress, others implement database structures for storing and locally organizing the data and others offer modules for applying well established meta-analysis algorithms and procedures. However, none of them allows altogether i) the batch retrieval and local organization of raw data files and of any related meta-information, ii) the re-annotation of samples to create user-defined batches of data, iii) the integrative analysis of data obtained from different platforms, and iv) the sharing of data, meta-information, analysis flows and results.

### A-MADMAN approach

The purpose of this paper is to present A-MADMAN, an open source web application for the meta-analysis of Affymetrix data contained in Gene Expression Omnibus (GEO). A-MADMAN allows retrieving, annotating, organizing and analyzing gene expression datasets. In particular, A-MADMAN addressees several of previously stated open issues in the meta-analysis of gene expression data allowing the integrative analysis of data obtained from different Affymetrix platforms through custom chip definition files and meta-normalization and the sharing of analysis flows and results.

## Implementation

### Architecture

A-MADMAN is a web application that allows retrieving gene expression datasets from GEO, annotating and locally organizing the downloaded samples, and generating an R object (*ExpressionSet*) which contains the integrated expression levels and all available metadata and sample characteristics. The gene expression data are obtained through a meta-analysis approach which includes signal generation, probe re-annotation into gene-centered identifiers, merging of expression levels from different experiments and a normalization step. A-MADMAN generates an *ExpressionSet *object in which the meta-expression levels from multiple experiments are completed by GEO-derived and user-defined metadata. The final *ExpressionSet *contains all the necessary information to perform, directly in R, any higher level analysis (e.g., SAM or limma) of all downloaded and integrated data.

**Figure 1:**
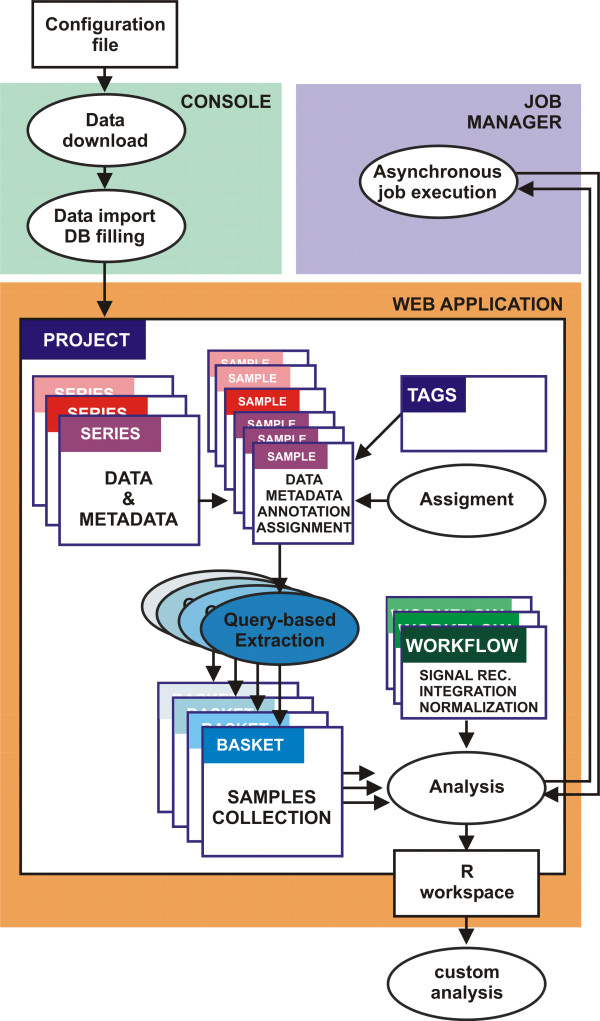
**A-MADMAN architecture**. A-MADMAN includes console, job server and web-application. The console allows data retrieval, import and database filling. The web application is the user friendly and collaborative core of the system allowing data inspection, annotation and analysis. A *Project is a *collection of samples, series, tags, baskets and analyses owned by a user or by a group of users. *Series *and samples data and metadata come from GEO. The user can create an annotation system based on tags and assign samples to individuals. Queries on Boolean combinations of annotation tags are used to select and extract groups of samples, giving rise to baskets. One or more baskets are passed to a customisable analysis workflow (a job server is in charge for asynchronous execution of jobs) outputting an R workspace, facilitating following analyses.

**Figure 2:**
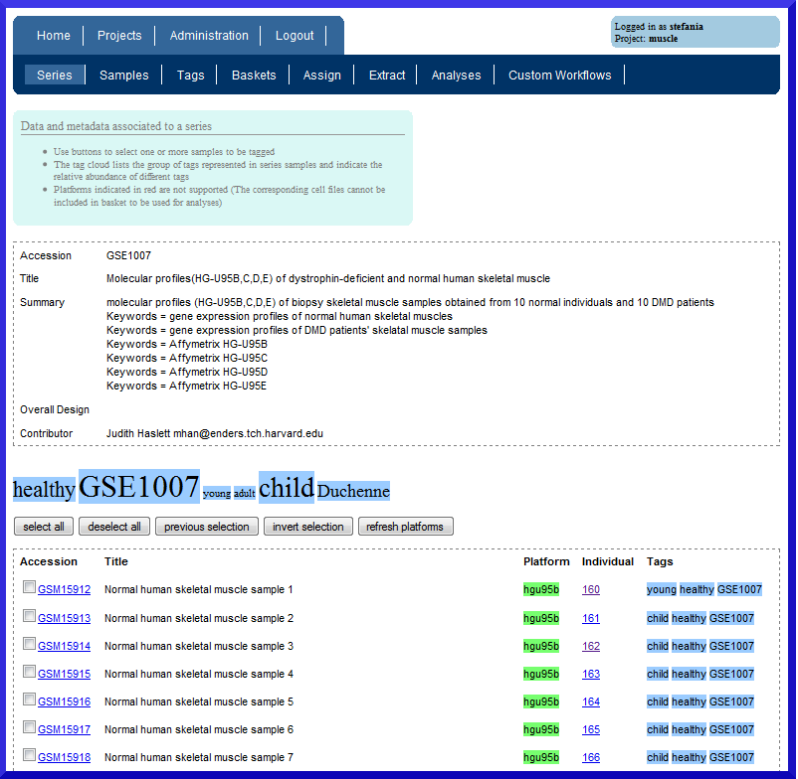
**Screenshot of the A-MADMAN web application**. The figure shows a series object, with associated data, metadata, user-defined annotation and sample assignment. A tag cloud indicates the relative frequency of tags associated to samples pertaining to the series.

A-MADMAN web application comprises a console, a job server and a web-application (Figure 1). The console is needed for the first phases of data retrieval, import and database filling. It performs the automatic download and organization, in a proper and transparent file system hierarchy, of raw data and annotations from Gene Expression Omnibus, starting from a configuration file listing the accession numbers of GEO series and/or samples to download. Metadata of GEO records are automatically imported into a local relational database to assist subsequent manual annotation and selection of samples from the web application. The job server is in charge for asynchronous execution of jobs which, depending on data size and algorithm, can be computationally intensive and take longer than allowed by an HTTP response-request cycle. The core of the framework is the web application, whose user friendly front-end facilitates data organization, annotation and analysis (Figure 2).

### Technical details

The software, written in python [22], is based on the popular Django web framework [23] and uses the R environment for statistical computing as a backend [24] [see Additional file 1]. A-MADMAN supports a collaborative working style for local or geographically dispersed teams through LAN or Internet deployment options, but can also be installed on a single personal computer with Windows operating system through an all-in-one package that bundles all required dependencies except R. The stand-alone package is completed by supporting material (i.e., program description and installation instructions) and by a step-by-step tutorial [25].

A-MADMAN natively supports retrieval and analysis of human data, but it can be customized, as explained in the documentation, for analyzing data from other species or to use third party custom CDF sets.

## Results

### Data organization

The *User *logins into the A-MADMAN web application with a personal account. A user may be part of a *Group *of users sharing the same rights on projects and related resources. A *Project *is a collection of samples, series, tags, baskets and analyses owned by a group. *Series *and samples are the primary data objects imported via the console commands. A *Sample *is a single experiment, each associated to a CEL file and to metadata extracted from the GEO *miniml *file. A *Series *is a collection of samples, as defined in GEO.

The user can create additional data entities, critical for planning and execution of analyses, and associate them to primary data. In particular, A-MADMAN supports the following entities:

*Individual*: a given biological entity (e.g., a patient) to which one or many samples refer to;

*Tag*: a free-text, descriptive label assigned to any sample and used for sample selection and grouping. The *tags *menu allows managing and creating tags to annotate/describe samples, through a labeling system whose complexity depends on the project, on the biological question and on the type of analysis.

*Basket*: a named collection of samples obtained making a logical query on tags.

### Data annotation

Data and metadata of selected *Series *and *Samples*, automatically downloaded from GEO, are imported into A-MADMAN and organized in a file system hierarchy. *Sample *characterization can be further enriched by user-defined annotations and tags. The tag based annotation system is central to A-MADMAN meta-analysis philosophy and can be used to:

i) exploit GEO metadata information, although incomplete, inaccurate and not conform to a controlled vocabulary. The A-MADMAN tag system allows the user to define his own vocabulary and consistency is enforced by the software itself (i.e. new tags must be defined and described before usage);

ii) test new hypotheses not envisioned by the original authors enriching the sample annotations with information derived from bibliographic references or from other sources.

**Figure 3:**
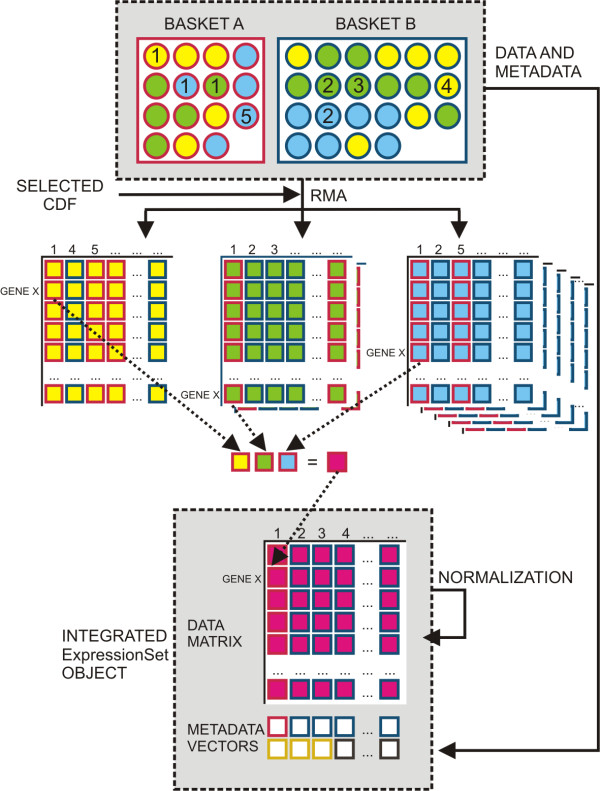
**Outline of the basic analysis workflow of A-MADMAN**. Once data and metadata are downloaded, organized and annotated, tag-based queries can be used to create data baskets. In the figure two data baskets are represented, each including samples obtained from three platforms (circles represent samples; red and blue borders represent two different baskets, corresponding for instance to healthy and diseased individuals; yellow, green ad light blue filling represent different platforms; numbers indicates specific individuals; squares stand for single expression values). RMA is carried out separately for each platform, according to the selected CDF, and gene expression levels and metadata, originating from user-defined annotations, are incorporated into a number of *ExpressionSet *objects corresponding to represented platforms. The annotations are used to generate a non-redundant list of gene-centered identifiers, which become the rows of a data matrix filled with integrated expression values, obtained averaging the expression levels of the various platform-specific probesets/metaprobesets (as shown for expression level of gene X in individual 1). After a final normalization step, by quantile distribution transformation, the integrated *ExpressionSet *object is produced.

*Samples *can be grouped in user-defined data baskets on the basis of logical queries performed on tag information (Figures 1 and 3). A parsing system has been implemented to specifically support this flexible extraction mechanism. The parser exploits a simple, custom query language where standard Boolean operators and explicit parenthesis precedence allow the user to populate baskets using a friendly interface and an almost natural language expression. For instance, once collected a muscle-related gene expression database and created a muscle physiology and disease tag system, a query like '*young AND male AND dystrophy AND NOT (Becker OR limb-girdle)*' allows populating a *Basket *with only those samples derived from young, male patients affected by any muscular dystrophy different from the Becker's or the Erb's ones.

*Baskets *can contain *Samples *from different platforms. Gene expression signals generation, platforms integration and normalization are then performed on *Baskets *according to the basic analysis workflow shown in Figure 3.

The labeling system allows creating data structures which are tailored to the subsequent meta-analysis. For instance, samples can be tagged to identify differentially expressed genes from two-class or multi-class comparisons or may be labeled to generate a large data matrix, accounting for different biological contests, when the goal is to detect co-expressed genes. Practically, the user can *Create a custom Tags system *and use it for *tagging *series/samples. The tag annotation system is based on the conceptual model of bottom-up classification, recently exploited by many social network web sites to organize and search data not amenable to enter a predefined and unambiguous taxonomy. This model enables the user to create a project-tailored, controlled, but unconstrained vocabulary to describe biological or technical characteristics of samples, which may be significant and informative according to the specific scopes of a meta-analysis.

One of the most critical problems in meta-analyses is originated by the inexplicit relationship among individuals/patients and samples. The granularity of Affymetrix-based gene expression data in GEO is represented by .CEL files but, depending on the platform, the RNA of a single individual may have been hybridized to more than one array, i.e. may be associated to more than a single .CEL file. A typical example of this one-to-many association between individuals and .CEL files is represented by the HG-U95 and HG-U133 sets with up to 5 or 2 possible raw data files for each biological sample, respectively, and a given .CEL file is not directly associated to a patient/sample ID. Said that, A-MADMAN interface considerably facilitate the matching of individuals to .CEL files and metadata, which can be inferred by combining metadata with information available in other formats and repositories, e.g. in a publication, in a supplementary information file, in a study-dedicated web site. A-MADMAN can relate each *Sample *to an *Individual *in two ways, i.e. through an automatic assignment (unique IDs, representing individuals, are given to samples) or using a knowledge-based assignment. In the latter case, the user-friendly interface allows matching samples and individuals according to the information derived from GEO or from original references or project websites.

### Dataset/s extraction

Once samples are tagged and assigned to individuals, the user may set up a query, based on a Boolean combination of annotation tags, to select and *Extract *samples and put them in a *basket*. The *baskets *represent the starting point of the subsequent analysis, i.e. the objects from which expression profiles are generated and information for the supervised comparisons are retrieved. Once selected one or more available baskets, all analyses will be conducted automatically, following a workflow selected by the user.

### Analyses

The action *Analyses *of A-MADMAN allows viewing analyses status, debugging information, logs and results, while the *Administration *action allows the administration of users, groups and projects, according to the allotted permissions. Analyses are conducted by the R backend powered by packages of the Bioconductor project [26], downloaded and installed automatically the first time they are required.

A-MADMAN contains a standard analysis workflow which may be applied to one or more baskets, but any *User *can create custom workflows, editing a workflow template, and share them within a *Group*. This feature makes easily customizable and expandable the analysis flow. The basic analysis workflow comprises the following steps:

1. creation of a work directory named after the current analysis and copy of the selected raw data files to the work directory;

2. signal reconstruction using RMA algorithm [27];

3. generation of an *ExpressionSet *containing expression profiles and metadata;

4. integration of different chip platforms trough matching of platform-dependent probesets to integrated probesets.

5. normalization of the final *ExpressionSet *with quantile distribution transformation [28].

The standard workflow is implemented as a template with three blocks of code that can be modified or replaced by the user, through the web interface, to define custom workflows. Specifically, the user can customize the *cdf_flavour*, i.e. the type of CDF used in the signal reconstruction (the default is GeneAnnot-based CDFs), the *signal_reconstruction*, i.e. the algorithm and parameters for signal reconstruction (the default is RMA with standard parameters), and the *additionalcode *block containing code to be run after the platform integration phase (the default performs the meta-normalization code). Blocks are written mixing R code [24] and some tags of the Django templating engine [23] and must conform to some conventions detailed in the A-MADMAN documentation. This type of customization, where the user can directly change the template code, allows a greater flexibility as compared to implementing a web interface with a fixed number of analytical options. The workflow customization is particularly useful in the frame of a collaborative resource, allowing easy sharing of both data and analysis schemas.

### Signal reconstruction

A-MADMAN supports different Affymetrix platforms and the integration of data obtained with subsequent generations of chips is achieved through the annotations of several, gene-centered CDF packages [see Additional file 2]. In a given meta-analysis project, a subset of supported platforms will be represented. The first step of analysis is the platform-specific quantification of expression levels from probes intensities. This can be carried out using RMA, probably the most efficient algorithm when dealing with the elevate number of Affymetrix arrays of a meta-dataset [27]. In this phase microarray probes can be re-organized and annotated using different Chip Definition Files [10,11]. In particular, for human expression data, the user may choose among standard Affymetrix CDFs [29], GeneAnnot-based CDFs [30] or Entrez-based CDFs [31].

If the data baskets under consideration are composed by samples obtained from more than one platform, RMA will be carried out separately for each platform on all those samples which are homogeneous with respect to the selected CDF (Figure 3). Gene expression levels and metadata, originating from GEO or from user-defined annotations, are then incorporated into an *ExpressionSet *object, i.e., into the Bioconductor class that contains and describes microarray expression level assays.

### Platforms integration

The annotations of the selected CDFs are used to generate a non-redundant list of gene-centered identifiers, each derived from one or more platform-specific probesets/metaprobesets. This list constitutes the backbone grid of the integrated data matrix whose values will be composed from gene expression signals derived from the various samples. Since each platform represented in the baskets will contribute expression data according to a list of platform-CDF dependent probesets (or metaprobesets), the values of the integrated data matrix will be obtained averaging the expression levels of the various platform-specific probesets/metaprobesets (Figure 2). It's worthwhile noting that if the original Affymetrix CDF is chosen, the expression values pertaining to a gene maybe averaged twice, i.e. intra and inter platform, given the probe redundancy inherent in these definition files.

According to the adopted CDF and to the combination of platforms in the basket, a fraction of the gene identifiers in the integrated data matrix may include some <NA> values. Since expression vectors including some <NA> values may represent a problem for the subsequent analyses, the user may choose to fill empty cells using a specific function, e.g. SAM imputes missing values via a K-Nearest Neighbor algorithm. In the case that a given gene is not represented in any platform of the baskets, i.e. if the data matrix has an entire row of <NA>, the record will be pruned so that the final integrated expression matrix contains only non-empty expression vectors.

Finally, the integrated data matrix is normalized to remove experiment-dependent biases. This normalization procedure is a crucial step to limit misleading outcomes due to the direct integration of different datasets. Among the various normalization techniques proposed for microarray meta-analysis, the quantile distribution transformation has been demonstrated to be highly efficient for normalizing data across experiments [28]. Data transformations based on a reference empirical distribution function, in fact, have been extensively applied on single channel microarray technologies showing their ability to increase inter-studies expression comparability. In this case, the median expression across the experiments with the largest available number of probesets has been used as the reference empirical distribution function in the quantile normalization.

After normalization, the integrated data matrix represents the observed expression levels of the final *ExpressionSet *object, while GEO-derived and user-defined metadata compose the *phenoData *slot. As such, the *ExpressionSet *contains all the necessary information to perform any higher level analysis (e.g., SAM or limma) directly in R.

### An example on muscle expression data

As a simple case study, we collected, annotated and analyzed with A-MADMAN a muscle-related gene expression dataset with the following main steps:

1. Data belonging to the GEO series GSE3307 (239 CEL files and related metadata) were downloaded and imported in a Project called "muscle-survey". The series belongs to the platforms GPL96 and GLP97 (Affymetrix HG-U133A and HG-U133B respectively), since most muscle biological samples were hybridized to both chips of the set.

2. Metadata and information coming from the original paper [32] were used to:

a) Build up a tag system, defining samples characterization (healthy, disease, age and disease type);

b) Apply the tags to the samples, obtaining an annotated database.

3. Samples were manually assigned to the corresponding individual IDs: Some individuals are represented two times in the samples (by two CEL files, one for each chip), since the same biological sample was hybridized to both platforms.

4. The query-based system was used to extract from the database three baskets:

a) A basket of 34 normal muscle samples for a total of 18 individuals;

b) Two baskets of Duchenne and Becker muscular dystrophy samples containing 20 and 9 samples respectively for a total of 15 individuals.

5. The default workflow (signal reconstruction, platform integration and normalization) was applied separately to the basket of normal samples and to the pair of Duchenne and Becker baskets, thus obtaining two ExpressionSets representing 11,174 GeneAnnot genes:

a) An ExpressionSet, collecting gene expression levels in normal muscle tissue, which can be exported and used for a classic co-expression analysis.

b) An ExpressionSet ready for the comparison of Duchenne and Becker expression profiles, for instance by SAM, to find genes differentially expressed in the two forms of the muscular dystrophy.

## Discussion

In recent years, high-throughput technologies represented a breakthrough for the analysis of gene expression levels and hundreds of microarray-based studies generated an overwhelming mass of potentially valuable data. Most of these data are organized in major repositories, are publicly available, and their meta-analysis represents an enormous opportunity for biologists and geneticists. However, the meta-analysis of data, obtained using a plethora of array platforms and experimental protocols in different laboratories, represents also a complex task for bioinformatics. Indeed, the integrated analysis of microarray data generated by different research groups on different array platforms requires *ad-hoc *computational methods to retrieve raw data files and any related meta-information, to fix incomplete, or otherwise inadequate, sample annotations, to integrate multiple data sets obtained using different technologies and to remove center- and platform-specific biases from the final integrated signals.

Despite major efforts to provide guidelines, formats, and standards for the annotation of microarray experiments, the identification, collection, and analysis of publicly available data sets still remains a cumbersome and error-prone process, further complicated by the heterogeneity of array designs and of sample characterization. The latter are among the most critical issues hampering meta-analysis approaches, since heterogeneous array designs complicate cross-platform integration and incomplete, or often inadequate, characterizations of specimens limit the robustness of statistical analyses. For the case of Affymetrix assays, the cross-platform comparison can partly be solved re-defining the array geometry through custom definition files and re-annotating the probesets in terms of unique entities (e.g., genes or transcripts). An expression profile associated to a unique gene identifier can be more easily matched across subsequent generations of microarrays [9-11] than a signal generated from a platform-dependent probeset. On the contrary, retrieval, organization and utilization of sample characterizations (i.e. the meta-information) are still critical and often severely limit the possibility to organize samples into biologically meaningful and homogeneous groups. Indeed, the relationship between specimen, biological sample, phenotypic characteristics and raw data files, the most granular object in repositories, may be not sufficiently explicit or, even worse, may be scattered around the web as supplementary data to a scientific manuscript. As such, procedures for efficiently associating large numbers of data files and their related meta-information may be extremely tedious and error prone [12].

Several software and web tools have been proposed for retrieval, organization and meta-analysis of microarray expression data from public repositories [13-21]. However, none of them offers the possibility to retrieve and organize both data and meta-information, to use the latter to re-annotate samples, and integrate data obtained from different platforms.

A-MADMAN is a novel tool for meta-analysis of Affymetrix data contained in Gene Expression Omnibus, which allows retrieving, annotating, organizing and analyzing gene expression datasets. In particular, A-MADMAN addressees i) the batch retrieval from Gene Expression Omnibus and the local organization of raw data files and of any related meta-information, ii) the re-annotation of samples, iii) the creation of user-defined batches of data through queries performed on sample labels, and iii) the integrative analysis of data obtained from different platforms through custom chip definition files and meta-normalization.

The batch retrieval of gene expression data from both GEO and Array Express can be carried out using WGAS [17], MaRe [20] and EMAAS [21] while EzArray [19], the GEOquery package of Bioconductor [18], the server version of SeqExpress [15] and the *GEOImporter *module of GenePattern [16] are limited to data deposited in GEO. In particular, the *GEOImporter *module of GenePattern allows retrieving only a single series at once and does not permit to perform sub-selections of samples. MaRe, EMAAS and WGAS can retrieve multiple series/datasets. However, MaRe is limited to the simple search and retrieval of data (i.e. no further local action is taken after retrieval), while the web centralization of WGAS poses serious operative limitations with very large datasets. A-MADMAN, once downloaded raw files and meta-information, locally organizes and displays the data through a user-friendly interface. Moreover, once data and metadata are retrieved, organized and annotated by A-MADMAN, they constitute a stable dataset which, being available through a web application, can be used to define and analyze different subsets of samples for answering different biological questions. The possibility of re-annotating samples, through user-defined assignments, is a peculiar feature of A-MADMAN and an improvement over all other available tools for meta-analysis. Indeed, a flexible assignment of .CEL files to specimens and a proper tracking of this association are necessary steps to efficiently exploit the mass of available data. However, handling multiple sample labeling and creating sub-classes of data may be so laborious and error-prone to discourage any meta-analysis diverse from the simple reproduction of the original experimental design. In A-MADMAN, samples are annotated using not only the original metadata but also a proprietary, controlled, but unconstrained vocabulary of descriptive terms. This project-tailored vocabulary allows adding biological, clinical, technical descriptions to the original sample annotations, thus widening the scopes of a meta-analysis. The annotation of samples through tags allows the definition and the extraction of any subset of data and, in principle, the meta-analysis of any user-defined experimental design. Specifically, one or multiple baskets of samples can be defined and extracted from the whole database simply using Boolean queries on the available tags and then used to construct any custom data matrix.

The platform integration step is not clearly addressed by most of the available tools. Indeed, the *ExpressionFileCreator *module of GenePattern, although allowing the use of custom CDF, cannot consider more than one platform at the time. Similarly, EzArray and EMAAS permit the analysis of only one type of Affymetrix platform in a given project. CrossChip [14], instead, uses probe by probe sequence comparison to integrate data from different generations of Affymetrix arrays. In CrossChip, when comparing Affymetrix platforms, only probes that have a certain amount of overlap in their nucleotide sequences in both arrays are retained in a *mask *file, which is later applied to the original .CEL files to generate new .CEL files. The new .CEL can be then be processed using any signal quantification algorithm. The major limitation of CrossChip is that only two platforms at the time can be compared. Differently, A-MADMAN can handle and integrate data generated by any type of Affymetrix arrays. Specifically, considering a set of samples obtained from more than one platform, expression signals are quantified separately for each platform on all those samples which are platform-homogeneous (Figure 3). Gene expression levels and metadata are incorporated into as many *ExpressionSet *objects as the different platforms represented in the baskets. The expression data of the various *ExpressionSet *(e.g., generated using RMA) are then integrated into a unique data matrix using a non-redundant list of gene-centered identifiers each derived from one or more platform-specific probesets/meta-probesets, using standard or custom CDFs. Each platform represented in the baskets contributes expression data according to a list of platform-CDF dependent probesets (or meta-probesets) and the values of the integrated data matrix are obtained averaging the expression levels of the various platform-specific probesets/metaprobesets (Figure 3). The final step of the integration is inter-platform quantile normalization, i.e. a data transformation using, as reference empirical distribution function, the median expression across the experiments with the largest available number of probesets.

A-MADMAN outputs an *ExpressionSet *object, which contains all the necessary information to perform any higher level analysis, i.e., the integrated data matrix of normalized expression levels and the GEO/user-defined metadata.

## Conclusion

We developed A-MADMAN a novel software which allows the retrieval, organization and meta-analysis of microarray expression data from public repositories. A-MADMAN presents several features not available in any current tool and specifically designed to plan and conduct meta-analyses of microarray expression data: i) perform the batch retrieval and local organization of raw data files and of any related meta-information, ii) re-annotate samples using meta-information, iii) create user-defined batches of specimens, and iv) integrate data obtained from different platforms through custom chip definition files and meta-normalization.

## Availability and requirements

• **Project name: **A-MADMAN

• **Project home page: **

• **Operating systems: **POSIX compliant and Win32 (developed on GNU/Linux)

• **Programming languages: **Python, R

• **Other requirements: **GNU R >= 2.8

• **License: **GNU GPL v3 or any later version

## List of abbreviations

GEO: Gene Expression Omnibus; CDF: Chip Definition File; HTTP: Hypertext Transfer Protocol; SOFT: Simple Omnibus Format in Text; SAM: Significance Analysis of Microarrays; NA: Not Available; miniml: MIAME Notation in Markup Language; MIAME: Minimum Information About a Microarray Experiment; RMA: Robust Multi-chip Analysis.

## Authors' contributions

AB planned, wrote and tested most of the software. AC contributed user interface code and participated to code review and quality control. FF participated to code review and algorithm planning, specifically for platforms integration. DR developed the inter-datasets normalization routine. SBo, CR, and SBi provided guidance during all phases of planning and implementing the software. AB, Sbo and Sbi wrote and revised the manuscript. All authors read and approved the final manuscript.

## Supplementary Material

Additional file 1 **A-MADMAN 1.4 source code**. Version 1.4 of A-MADMAN source code. Click here for file

Additional file 2 **Supplementary Figure 1**. The role of raw data-to-individuals assignment in the process of platforms integration. Click here for file
