# Supplementary material for: A-MADMAN: Annotation-based microarray data meta-analysis tool
Source: BMC Bioinformatics. 2009 Jun 29;10:201. doi: 10.1186/1471-2105-10-201 (PMC2711946; doi:10.1186/1471-2105-10-201)
Supplement: Additional file 1 — A-MADMAN 1.4 source code. Version 1.4 of A-MADMAN source code. [file 1471-2105-10-201-S1.zip › amadman/ua_manager/media/docs/linux-installation.html]

A-MADMAN docs (GNU/Linux installation)


# A-MADMAN installation on GNU/Linux (recommended choice for LAN/WAN deployment)

## Dependencies:

We tried to keep external dependencies at minimum:   
You will need:   

- Python >= 2.5
- Django >= 1.0
- GNU R >= 2.8
- A relational DataBase (mysql is our choice for our deployment but any RDBMS supported by django should work)
- The corresponding python db module (PyMySQLdb for example if you choose mysql)

For ubuntu 9.04 this command should install all necessary dependencies (using R official binary repositories to obtain the latest R version):

apt-get install python2.6 mysql-server python-mysqldb python-django r-recommended r-base-dev

On Red Hat derivatives you should install the packages: python MySQL-python mysql django and the R binary packages from R repositories

## Installation and configuration

- Download the last version of A-MADMAN source package and extract it where you prefer.
- Create an empty database for A-MADMAN.
- open a terminal and go where you extracted the source.
- copy the file localconf-skeleton.py as localconf.py
- edit localconf.py to customize the settings the file content looks like this:

```
NUAM_HOME=""
DATA_HOME=""
WORK_HOME=""
DEBUG = False
TEMPLATE_DEBUG = DEBUG
DATABASE_ENGINE = ''           # 'postgresql_psycopg2', 'postgresql', 'mysql', 'sqlite3' or 'ado_mssql'.
DATABASE_NAME = ''             # Or path to database file if using sqlite3.
DATABASE_USER = ''             # Not used with sqlite3.
DATABASE_PASSWORD = ''         # Not used with sqlite3.
DATABASE_HOST = ''             # Set to empty string for localhost. Not used with sqlite3.
DATABASE_PORT = ''             # Set to empty string for default. Not used with sqlite3.
MEDIA_URL = 'http://yourhost.yourdomain/media'
R_BIN=''
DEVEL_BASE_URL = ''
LOG_DIR='/tmp'     
ADMIN_MEDIA_PREFIX='/admin_media/'
CACHE_BACKEND='db://cache'
CACHE_TIME=60*60
```

- *NUAM\_HOME* is the path where you extracted the A-MADMAN sources.
- *DATA\_HOME* is the path where data from GEO will be stored in the filesystem.
- *WORK\_HOME* is the path where the analyses working directories will be created.
- *DEBUG* enables extensive debugging info when something goes wrong (potentially unsafe for Internet deployment) Use only with django development server.
- *TEMPLATE\_DEBUG* enables template debugging.
- *DATABASE\_\** Database connection settings.
- *MEDIA\_URL* base url serving static media stuff.
- *ADMIN\_MEDIA\_PREFIX* prefix of url serving administration static files
- *R\_BIN* path to the GNU R binary.
- *DEVEL\_BASE\_URL* dummy option for serving static media files when running the django development server
- *LOG\_DIR* path where log files generated by geoget and by the job server are saved

- To syncronize your database with the A-MADMAN schema issue this command in the A-MADAMN directory:

```
python manage.py syncdb
	  python manage.py createcachetable cache
```

You'll be asked to create a superuser; answer yes and fill in the required information. You'll use this user to log in the first time.

## Test

Once set the appropriate variables you can test your installation with the django development server:

- Temporarily set DEBUG=True and DEVEL\_BASE\_URL='http://localhost:8000/amadman' in localconf.py
- issue the command:

python manage.py runserver

- Point your browser to http://localhost:8000/amadman/

## Deployment

First set DEBUG=False in localconf.py
The preferred way to deploy a Django application for LAN/Internet use is behind apache/mod\_python.

On ubuntu 9.04 you can install the necessary stuff with:

```
apt-get install apache2 apache2-mpm-prefork libapache2-mod-python
```

On Red Hat derivatives you should install httpd and mod\_python

Then you have to configure mod\_python.   

On ubuntu 9.04 you can write a python.conf file in /etc/apache2/conf/

On Red Hat derivatives you should write a python.conf file in /etc/httpd/conf.d/

The following should work (assuming you installed A-MADMAN on /usr/local/).

```
<Location "/amadman">
   SetHandler python-program
   PythonPath "['/usr/local','/usr/local/amadman'] + sys.path"
   PythonHandler django.core.handlers.modpython
   SetEnv DJANGO_SETTINGS_MODULE amadman.settings
   PythonInterpreter amadman
   PythonDebug On
 </Location>

 <Location "/media">
  SetHandler None
 </Location>

 <Location "/admin_media">
  SetHandler None
 </Location>
```

We'll serve static media files (css,image,etc..) from apache without the intervention of mod\_python and django.   
So we create a pair of symbolic links (same path assumptions as before).

On ubuntu 9.04:

```
$ sudo ln -s /usr/local/amadman/ua_manager/media/ /var/www/

	  $ sudo ln -s /usr/share/python-support/python-django/django/contrib/admin/media/  /var/www/admin_media
```

On Red Hat derivatives:

```
# ln -s /usr/local/amadman/ua_manager/media/ /var/www/html/
	  # ln -s $(DJANGO_ROOT)/django/contrib/admin/media/ /var/www/html/admin_media
```

In localconf.py change the settings:

- MEDIA\_URL='http://yourhost.yourdomain/media'
- ADMIN\_MEDIA\_PREFIX='/admin\_media/'

(values must be different if you choose different symbolic link names)


Restart your web server and point you browser to http://yourhost.yourdomain/amadman/ and login with the credentials you entered when you initialized the database.
Use the Administration Interface to create at least a group and a project. Name them both *test* if you want to be able to follow the tutorial for windows user

To have some data to play with:

```
python manage.py geoget --georc georc.example
    python manage.py geotodb --georc georc.example --project test
```

To start the job server type the command:

```
python manage.py mtasyncd --start
```

To stop it run:

```
python manage.py mtasyncd --stop
```

You can follow the windows tutorial to explore A-MADMAN keeping in mind to issue every text command in a server terminal instead of clicking on the start shortcuts...

## Troubleshooting

You could encounter permissions issues.
In particular:

- the path specified in DATA\_HOME must be writeable by the user under wich you run the geoget command
- the path specified in WORK\_HOME must be writeable by the user running the job server
- the directory where R installed packages downloaded from the internet must be writeable by the user running the job server (in ubuntu it is /usr/local/lib/R/site-library/ )
