# Supplementary material for: A-MADMAN: Annotation-based microarray data meta-analysis tool
Source: BMC Bioinformatics. 2009 Jun 29;10:201. doi: 10.1186/1471-2105-10-201 (PMC2711946; doi:10.1186/1471-2105-10-201)
Supplement: Additional file 1 — A-MADMAN 1.4 source code. Version 1.4 of A-MADMAN source code. [file 1471-2105-10-201-S1.zip › amadman/ua_manager/media/docs/tutorial.html]

A-MADMAN docs (tutorial for windoz users)


# Tutorial (for Windows Users)

## Installation

1. Install the latest release of GNU R (at least 2.8).
2. Download the latest A-MADMAN all-in-one package
3. Click on A-MADMAN-setup.exe and follow the instructions in particular:

- It's mandatory to choose an installation directory where you have permissions to write (the default is the User Document Folder).
- A-MADMAN tries to guess Rterm.exe (R command line interpreter) path looking in the registry. Change the default only if you are sure the guess is wrong.

## Technical details (skip if you don't care)

The all-in-one package is quite big because for ease of deployment bundles a few software packages:

- An almost complete Python 2.5 distribution (Powerful OO interpreted programming language).
- Django 1.0 (Popular Python web framework).
- CherryPy 3.1 (A pure python multithreaded web server, because django development server is not recommended for production use)
- A-MADMAN itself and its dependencies: urlgrabber, ply.

All important settings are stored in localconf.py   
For the all-in-one package we choose reasonable defaults.
In particular we provide a SQlite db preconfigured with necessary tables and default administrative data preloaded.
Feel free to change the defaults...

## Concepts and terminology

- *User*:user of A-AMADMAN system can be part of many groups.
- *Group*: named group of users who share the same rights on projects (and related resources).
- *Project*: a named collection of samples, series, tags, baskets and analyses owned by a group.
- *Sample*: a single experiment (with an associated CEL file).
- *Series*: a collection of samples (as in GEO).
- *Individual*: a patient to which one or many samples refer.
- *Tag*: a free text descriptive tag assigned to samples used for samples selection.
- *Basket*: a named collection of samples obtained making a logical query on tags.

The all-in-one package creates a user named *admin* with password *admin* (as the name suggest can also access the administrative interface).
A default group named *test* is also created. The test groups owns the default project named also *test*. (*admin* is obviously part of group *test*)

## Data Retrieval

Data to retrieve must be specified in a configuration file. The syntax is pure python. You define a simple data structure that specifies the names of series and samples to download.   
In the installation directory there is an example configuraton file named georc.example.

```
data={'GSE1004': {'samples': ['GSM15807',
                         'GSM15822',
                         'GSM15823',
                         'GSM15824',
                         'GSM15825',
                         'GSM15826',
                         'GSM15827',
                         'GSM15828',
                         'GSM15829',
                         'GSM15830',
                         ]},
      
      'GSE1786': {'samples': ['GSM30842',
		             'GSM30843',
			     'GSM30844',
			     'GSM30836',
			     'GSM30837',
			     'GSM30838',
		]},
  }
```

You can specify the names of samples if you need only a subset of samples from a series or 'all' if you need all for example:

```
data={'GSE1004': {'samples': 'all'},
      'GSE1786': {'samples': 'all'}
  }
```

Launch the A-MADMAN console from the Start Menu and start the download process with:

python manage.py geoget --georc georc.example

The download process will take a while... (depending on how many series you selected and how fast is your network).   
If something goes wrong reissue the command and the process will restart from where it left.

## (Meta) Data import

In the A-MADMAN console run this command:

python manage.py geotodb --georc georc.example --project test

This command will create series and samples objects in the database and will import the associated metadata (in the *test* project).

## Login and first look

From the Start Menu launch start-amadman and point your favourite browser to http://localhost:8000/amadman/.   
In the main menu on the top of the page click projects.   
A login form will appear. Login with username *admin* and password *admin*.   
Then from the projects drop down menu select the *test* project (the only one).   
Now the complete menu is available:

- Series (series overview and details).
- Samples (samples overview and details).
- Tags (manage and add new tags to annotate/describe samples).
- Assign (assignment interface to assign samples to individuals).
- Extract (select samples issuing a query on tags and put them in a basket ).
- Baskets (manage baskets, select baskets to analyse together and select workflow for the analysis).
- Analyses (view analyses status, debug info, logs and download results ).
- Custom Worflows (create a custom workflow editing the base workflow template).
- Administration (administer users, groups and projects).
- Logout

## Annotation of samples

### Tag creation

We'll create three tags to annotate our samples.   
Click Tags in the main menu and add three tags using the form.   
Name them: young, old and sedentary (a brief description is mandatory).   

### Adding tags to samples

Now you can use the tags to annotate the samples.  
  
Start with series GSE1004:

- click the *select all* button select *young* from the tags multiple selection box and click *add*.

Now go on with series GSE1786:

- click the *select all* button select *old* from the tags multiple selection box and click *add*.
- mark the samples GSM30842, GSM30843, GSM30844 clicking the check box next to the sample name, then select *sedentary* from the tags box and press *add*.

## Baskets creation

Click *Extract* on the top menu.   
The interface for query creation is shown.   
Click on *young* and the press the *preview* button.   
In a new tab/window you can preview the results of this simple query (all samples tagged with *young*)   
Now close the tab/window and press *go*   
Now give a name (name it *'youngs'* ) and description to the newly created basket and press the *save* button.

Click again *Extract* and compose the following query:   
*'old and not sedentary'*   
Preview the query results.
Then create and save a new basket and name it *'well\_trained'*

## Individuals assigment

In this tutorial we will proceed with a rapid automatic assignment: a new individual is assigned to each sample. In a real meta-analysis you would have to check carefully if more samples refer to the same individual and annotate properly this situation. See here for detailed instructions.   
Click Assign in the top menu choose a series clicking on the number of samples left to assign and then press the auto button.
Do the same for the other series.

## Vanilla Analysis

Click on *Baskets* on the top menu.   
Mark the two baskets you just created *youngs* and *well-trained* and press the *create analysis* button.   
Name the analysis *'test1'*, leave the default and only workflow *vanilla* and press the *save* button.

The analysis is now queued for execution and must be executed by another a-madman component the *job server*.   
Select start-job-server from Start Menu -> A-MADMAN   
The job server will continue to run until the window is closed, waiting for analyses to execute.

The analysis status will change from waiting, to running to done.   
When the status is done you can click on the analysis name and see the execution log, download the R workspace and see the generated R code.

## Defining a custom workflow

We'll define a workflow to substitute rma signal reconstruction with gcrma.

Click *Custom WorkFlows* on the top menu and press the *new* button.

Give the new workflow the name *gcrma*, fill in a brief description and substitute the default text in the template field with the following code and press the *save* button

```
{% extends "basic.rtmpl" %}
  {% load R %}

  {% block  cdf_flavour %}
    flavour="affy"
  {% endblock %}

  {% block signal_reconstruction %}
  load_or_get_from_bioc("gcrma")
  {% for chip_name in chip_names %}
    load_or_get_from_bioc("{{chip_name}}probe")
    ai <- compute.affinities("{{chip_name}}")
    eset.{{chip_name}} <- gcrma(batch.{{chip_name}},affinity.info=ai)
  {% endfor %}   
  {% endblock %}

  {% block additionalcode %} 
    load_or_get_from_bioc("preprocessCore")
    ieset=metanorm(ieset)
  {% endblock %}
```

In this case we changed only the *signal reconstruction* entry point and leaved the rest of the workflow unaltered. See the general documentation for details.

## Running an analysis with a custom workflow

Click on *Baskets* on the top menu.   
Select the two baskets and press the *create analysis* button.   
Give a name to the newly created analysis, select *gcrma* from the Workflow drop menu and press the *save* button.
