# Supplementary material for: A-MADMAN: Annotation-based microarray data meta-analysis tool
Source: BMC Bioinformatics. 2009 Jun 29;10:201. doi: 10.1186/1471-2105-10-201 (PMC2711946; doi:10.1186/1471-2105-10-201)
Supplement: Additional file 1 — A-MADMAN 1.4 source code. Version 1.4 of A-MADMAN source code. [file 1471-2105-10-201-S1.zip › amadman/ua_manager/media/docs/general.html]

A-MADMAN docs


# Introduction

A-MADMAN is a open source web application and gene chip analysis automation framework written in python with a focus on meta-analysis of data published on public repositories (only GEO is supported at moment).
It's based on the popular Django web framework and uses GNU R as a backend.

A-MADMAN tries to automate many tedious and error prone steps an investigator has to accomplish to setup a working environment for conducting meta-analyses without giving up power and flexibility.

A-MADMAN supports a collaborative working style for local or geographically dispersed teams through LAN or Internet deployment options (GNU/Linux recommended), but can be used also by a single researcher on his Windows® Personal Computer installing an all-in-one package that bundles all required dependencies except R.

# Motivation

Conducting meta-analyses of public available data can be a daunting task for many reasons:

- Incomplete Annotation of datasets.
- It can be hard to track the relationship beetwen patient, biological samples and CEL files (the most granular data object in repositories).
- Managing a large number of data files and related meta data can be very tedious and error prone and can limit the reproducibility of analyses.
- Software to analyse microarray data is widely available and very good open source implementations can be obtained for free but can be cumbersome to use and require some administrative overhead:

- Need to manually install CDF and annotation packages for every platform you intend to take into consideration (at least if you prefer to use custom CDFs).
- Prepare a working directory for conducting the analysis and storing the results.
- No easy way to handle datasets produced with different chip platforms.
- No easy way to share the work with coworkers and others.

# Features

A-MADMAN aims to lower the bar for starting a meta-analysis study offering these features:

- Automatic download and organization in a proper and trasparent filesystem hierarchy of GEO raw data and annotations given a simple configuration file.
- Automatic import of meta data of GEO records into a local relational database to assist subsequent manual annotation and selection of samples.
- A flexible annotation system based on tags (a la web 2.0).
- A user friendly Assignment interface to assist the user in matching samples to individuals. (patient or equivalent statistical unit).
- Samples to analyse together are selected with an arbitrary complex logical query on tags for example: 'young and dystrophy and not (becker or limb-girdle)' and placed in a named object called basket.
- Analyses are conducted by the R backend powered by packages of Bioconductor project (downloaded and installed automagically the first time they are required).
  A basic workflow (named 'vanilla') is provided that comprise these steps:
  - Creation of a work directory named after the analysis.
  - Copying of the selected CEL files to the work directory.
  - Signal reconstruction using RMA algorithm and Ferrari et al. custom CDFs.
  - Transfer of metadata to Bioconductor ExpressionSet objects
  - Integration of different chip platforms using precalculated 'masks' based on chip annotations.
  - Pruning of '<NA> only' probesets in the final expression matrix.
  - Normalization of final ExpressionSet with distribution transformation.

In the following image a sketch representing platform integration intricacies is shown:

# Usage notes

Assumptions for the following notes are

- You are running A-MADMAN (properly configured) on a GNU/Linux server and accessing it via a web browser on a generic client machine.
- You have shell access to the server and write permissions on the directory where data are downloaded. (refer to linux-installation for configuration details)

If you plan to use the standalone all-in-one package please follow the tutorial for windows users instructions instead.

## Data Retrieval

Data to retrieve must be specified in a configuration file. The syntax is pure python. You define a simple data structure that specifies the names of series and samples to download.

Syntax example:

```
data={'GSE1004': {'samples': ['GSM15807',
                         'GSM15822',
                         'GSM15823',
                         'GSM15824',
                         'GSM15825',
                         'GSM15826',
                         'GSM15827',
                         'GSM15828',
                         'GSM15829',
                         'GSM15830',
                         ]},
      
      'GSE1786': {'samples': ['GSM30842',
		             'GSM30843',
			     'GSM30844',
			     'GSM30836',
			     'GSM30837',
			     'GSM30838',
		]},
  }
```

You can specify the names of samples if you need only a subset of samples from a series or 'all' if you need all.

To start the download process log in to the server and issue from the shell the following command (in the directory where A-MADMAN is installed):

python manage.py geoget --georc yourconfigurationfile.georc

The download process will take a while... (depending on how many series you selected and how fast is your network).   
If something goes wrong reissue the command and the process will restart from where it left.

## (Meta) Data import

In the server shell (in the directory where A-MADMAN is installed) run :

python manage.py geotodb --georc georc.example --project projectname

This command will create series and samples objects in the database and will import the associated metadata (in the *projectname* project).

The project must already exists and to be able to manipulate data later in the web application your user has to be a member of the group which owns the project. Define your own policies with the *Administration* web interface

## Assignment

To analyse the samples A-MADMAN needs to know which samples refer to the same patient (or cell line or whatever).

An individual in A-MADMAN is represented simply as a numerical identifier

In the trivial case each sample corresponds to a different individual.

In this case on the series page (assignment is done at the series level for each sample) you can click the link *assign* of an unassigned sample to reach the Assignment interface.
Here you can press the *auto* button and each unassigned sample of the series will be assigned to a newly created individual.

In a typical situation more samples (corresponding to different cel files) will refer to the same individual.

To assign a sample click its *assign* link.

To reduce the probability of errors you are not prompted to fill the individual field.
Instead if the sample you are assigning refers to an individual not previously seen press the *new* button and a new individual will be created for you.

Otherwise if the sample refer to an individual you saw before click on the individual number link (notice that the individual field near the sample name will be automatically filled) and press save.

To identify more easily samples referring to the same individual exploiting the sample title field you can filter for specific title words filling the *Filter by title* field and pressing the *filter* button.

See the image below to have a glimpse of how assigment information is crucial for production of a an integrated expression matrix. (click on the image for full resolution)

# Custom Workflows

A workflow is defined as a django template that gets some python variables in input from the web application and generates at runtime R source code to conduct the analysis. We provide a basic workflow that exposes some entry points to customize the analysis.
This is the default workflow:

```
{% extends "basic.rtmpl" %}
{% load R %}

{% block  cdf_flavour %}
   flavour="ferrari"
{% endblock %}

{% block signal_reconstruction %}
 {% for chip_name in chip_names %}
   eset.{{chip_name}} <- rma(batch.{{chip_name}})
 {% endfor %}
{% endblock %}


{% block additionalcode %}
 ieset=metanorm(ieset)
{% endblock %}
```

The standard template itself extends a more basic template (basic.rtmpl) which implements the code needed to

- download on demand CDF and annotation packages.
- integrate data coming from different platforms.
- propagate sample metadata from python classes to Bioconductor classes.

We provide three entry points to customize things:

- *cdf\_flavour*

Here you can choose a different set of CDFs currently implemented alternatives are:

- 'affy': the official affymetrix CDFs.
- 'dai:' Dai et al. Nucleic Acid Research 33 (20), e175 (entrez version)

- *signal\_reconstruction*

Here you can change the way signal reconstruction is performed.
You have at this point a vector listing the chip platform names *chip\_names* and a batch.*chipname* AffyBatch object for every platform represented in the samples being processed.
You must provide an ExpressionSet object named eset.*chipname* for every platform.

- *additionalcode*

Now in the workspace are available *iemat* (integrated expression matrix) and *ieset* (integrated ExpressionSet).
The default code is this block apply to *ieset* a distribution transformation (quantile normalization) implemented in the rnuam R package hosted on our R repository.
You can add to this block your own code to do whatever you like.   
Remember to add the names of the objects you create to objects\_to\_save vector to let amadman save them for you in the workspace.
For example:

```
{% block additionalcode %}
 ieset=metanorm(ieset)
 stuff=do_some_stuff_with_ieset(ieset)
 objects_to_save=c(objects_to_save,"stuff")
{% endblock %}
```

# Security Notes

**Users are trusted by default** and the code they inject in custom workflows is not checked for filtering malicious instructions.
The code is passed as it is to the job server and executed with the privileges of the user under which is running.

# Analyses freshness

Baskets are 'lazy evaluated' when needed i.e. the samples that satisfy the query are updated when needed.
Instead an analysis is a still image of how the baskets looked like when it was run.
So can become 'stale' if you add or remove tags that appear on the query.
To check if the analysis refers to the current definition of its baskets ('fresh' in A-MADMAN terminology) press the *check freshness* button on the Analyses page.

# Customize A-MADMAN for organisms other than Homo Sapiens.

While giving general instructions we'll walk through a practical example to support a pair of rat chips (Affymetrix Rat Expression Set 230 and 230 2.0).  
The OS of reference for the example is GNU/Linux.

- Add entries for new chips to support to the variable SUPPORTED\_CHIPS in settings.py

```
SUPPORTED_CHIPS=(
    'hgu95av2',
    'hgu95b',
    'hgu95c',
    'hgu95d',
    'hgu95e',
    'hgu133a',
    'hgu133b',
    'hgu133plus2',
#new from here
    'rae230a', 
    'rae230b', 
    'rat2302'  
    )
```

- Add entries to the variable CHIP\_META\_DATA in settings.py. The field siblings lists the other chips of the same set.

```
CHIP_META_DATA={
    'hgu95av2': {
        'siblings':['hgu95b','hgu95c','hgu95d','hgu95e']
        },
    'hgu95b': {
        'siblings':['hgu95av2','hgu95c','hgu95d','hgu95e']
        },
    'hgu95c':{
        'siblings':['hgu95av2','hgu95b','hgu95d','hgu95e']
        },
    'hgu95d':{
        'siblings':['hgu95av2','hgu95b','hgu95c','hgu95e']
        },
    'hgu95e':{
        'siblings':['hgu95av2','hgu95b','hgu95c','hgu95d']
        },
    'hgu133a':{
        'siblings':['hgu133b']
        },
    'hgu133b':{
        'siblings':['hgu133a']
        },
    'hgu133plus2':{
        'siblings':[]
        },
# new from here
    'rae230a':{
        'siblings':['rae230b']
        },
    'rae230b':{
        'siblings':['rae230a']
        },
    'rat2302':{
        'siblings':[]}
}
```

- Make sure that CDF and annotation packages, for the chips you want to support, are available in a R compatible software repository. Otherwise install them manually or build youself a repository.

- Choose a name for the new flavour that will identify from now one the combination of newly supported chips and selected CDF. The flavour name will be used for mask preparation and for the custom workflow definition. For our example we choose "affyrat" as flavour name.

- Prepare an R source file and implement some functions to describe details about the flavour A-MADMAN must know to prepare the mask for platform integration and to analyze your data. This step is necessary because different custom CDF and annotation packages use different naming conventions and repositories. So to remain general we must specify a great deal of details.

We'll call our example file rats.R

- get\_flavour\_cdf\_names

```
# given chips names in bioconductor convention return names of cdf packages
	    get_affyrat_cdf_names <- function(chips)
            {
              return(paste(chips,"cdf",sep=""))
            }
```

- get\_flavour\_annotations\_names

```
#  given chips names in bioconductor convention return names of annotations packages
	    get_affyrat_annotations_names <- function(chips)
            {
              return(paste(chips,".db",sep=""))
            }
```

- get\_flavour\_index\_column

```
#return what column contains the unique identifier for inter-platform matching
	    get_affyrat_index_column <- function()
            {
              return("ENTREZID")
            }
```

- get\_flavour\_repo

```
#return url of R compatible software repository hosting CDFs and annotations packages
	    get_affyrat_repo <- function()
            {
              return("bioc")
            }
```

- get\_flavour\_mask\_repo

```
#return  url of R compatible software repository that will host flavour specific mask
	    get_affyrat_mask_repo <- function()
            {
              return("http://compgen.bio.unipd.it/R")
            }
```

- get\_flavour\_type

```
#return either "affy"  or "not affy". Original CDF and custom CDF are processed differently
	    get_affyrat_type <- function()
            {
              return("affy")
            }
```

- package\_to\_annotation\_flavour\_name\_mangler

```
#given name of annotation package returns base name of annotation objects
	    package_to_annotation_affyrat_name_mangler <- function(name)
            {
              mangled <- sub(".db","",name)
              return(mangled)
            }
```


- Build a mask package for your flavour using the package rnuam in R.

  If you ran an analysis with A-MADMAN you should already have it. Otherwise install it within R with:

  ```
  >install.packages("rnuam",repos=c("http://compgen.bio.unipd.it/R"))
  ```

  Inside R prepare the mask package.   
  Use function make\_affy\_masks for oficial CDFs and make\_masks\_not\_affy for custom CDFs.  
  Both take the same arguments:

  - flavour: name of the flavour
  - basedir: path where the mask package source dir will be created
  - chips: vector of chips included in the mask

  ```
  > library(rnuam)
  	    > source("rats.R")
  	    > make_affy_masks(flavour="affyrat",basedir="/home/bisio/r-chip-tests/",chips=c("rae230a","rae230b","rat2302"))
  ```
- Build the mask package from the shell:

```
bisio@ada:~/r-chip-tests$ R CMD build affyratmasks
      * checking for file 'affyratmasks/DESCRIPTION' ... OK
      * preparing 'affyratmasks':
      * checking DESCRIPTION meta-information ... OK
      * removing junk files
      * checking for LF line-endings in source and make files
      * checking for empty or unneeded directories
      * building 'affyratmasksv2.9_47.0.tar.gz'
```

- make it available on a R compatible software repository or install it on the machine hosting A-MADMAN

```
bisio@ada:~/r-chip-tests$ R CMD INSTALL affyratmasksv2.9_47.0.tar.gz 
      * Installing to library '/opt/R-2.9.0/lib64/R/library'
      * Installing *source* package 'affyratmasksv2.9' ...
      ** data
      No man pages found in package  'affyratmasksv2.9'
      ** building package indices ...
      * DONE (affyratmasksv2.9)
```

- Create a new custom workflow in A-MADMAN. In the cdf\_flavour block you'll specify your new flavour name and the code that you used to build the mask. In our example we'll call it rats

```
{% block  cdf_flavour %}
   flavour="affyrat"
   get_affyrat_cdf_names <- function(chips)
   {
    return(paste(chips,"cdf",sep=""))
   }

   get_affyrat_annotations_names <- function(chips)
   {
    return(paste(chips,".db",sep=""))
   }

   get_affyrat_index_column <- function()
   {
    return("ENTREZID")
   }

   get_affyrat_repo <- function()
   {
    return("bioc")
   }

   get_affyrat_mask_repo <- function()
   {
    return("http://compgen.bio.unipd.it/R")
   }

   get_affyrat_type <- function()
   {
    return("affy")
   }
  
   package_to_annotation_affyrat_name_mangler <- function(name)
   {
    mangled <- sub(".db","",name)
    return(mangled)
   }
{% endblock %}
```

- Now you can analyze your data with the custom workflow you created.
