# Supplementary material for: A-MADMAN: Annotation-based microarray data meta-analysis tool
Source: BMC Bioinformatics. 2009 Jun 29;10:201. doi: 10.1186/1471-2105-10-201 (PMC2711946; doi:10.1186/1471-2105-10-201)
Supplement: Additional file 1 — A-MADMAN 1.4 source code. Version 1.4 of A-MADMAN source code. [file 1471-2105-10-201-S1.zip › amadman/ua_manager/templates/admin/base.html]

{% block title %}{% endblock %}

{% if LANGUAGE\_BIDI %}{% endif %}
{% block extrastyle %}{% endblock %}
{% block extrahead %}{% endblock %}
{% block blockbots %}{% endblock %}
{% load i18n %}

{% if not is\_popup %}

{% block branding %}{% endblock %}

{% if user.is\_authenticated and user.is\_staff %}

{% trans 'Welcome,' %} **{% if user.first\_name %}{{ user.first\_name|escape }}{% else %}{{ user.username }}{% endif %}**. {% block userlinks %}{% url django-admindocs-docroot as docsroot %}{% if docsroot %}{% trans 'Documentation' %} / {% endif %}{% trans 'Change password' %} / back to A-MADMAN / {% trans 'Log out' %}{% endblock %}

{% endif %}
{% block nav-global %}{% endblock %}

{% block breadcrumbs %}

{% trans 'Home' %}{% if title %} › {{ title|escape }}{% endif %}

{% endblock %}
{% endif %}
{% if messages %}

{% for message in messages %}- {{ message|escape }}
{% endfor %}
{% endif %}

{% block pretitle %}{% endblock %}
{% block content\_title %}{% if title %}

# {{ title|escape }}

{% endif %}{% endblock %}
{% block content %}
{% block object-tools %}{% endblock %}
{{ content }}
{% endblock %}
{% block sidebar %}{% endblock %}

{% block footer %}{% endblock %}
