# Supplementary material for: A-MADMAN: Annotation-based microarray data meta-analysis tool
Source: BMC Bioinformatics. 2009 Jun 29;10:201. doi: 10.1186/1471-2105-10-201 (PMC2711946; doi:10.1186/1471-2105-10-201)
Supplement: Additional file 1 — A-MADMAN 1.4 source code. Version 1.4 of A-MADMAN source code. [file 1471-2105-10-201-S1.zip › amadman/ua_manager/templates/admin/base_site.html]

{% extends "admin/base.html" %}
{% load i18n %}
{% block title %}{{ title }} | {% trans 'A-MADMAN admin' %}{% endblock %}
{% block branding %}

# {% trans 'A-MADMAN administration' %}

{% endblock %}
{% block nav-global %}{% endblock %}
