# Supplementary material for: A-MADMAN: Annotation-based microarray data meta-analysis tool
Source: BMC Bioinformatics. 2009 Jun 29;10:201. doi: 10.1186/1471-2105-10-201 (PMC2711946; doi:10.1186/1471-2105-10-201)
Supplement: Additional file 1 — A-MADMAN 1.4 source code. Version 1.4 of A-MADMAN source code. [file 1471-2105-10-201-S1.zip › amadman/ua_manager/templates/admin/index.html]

{% extends "admin/base\_site.html" %}
{% load i18n %}
{% block stylesheet %}{% load adminmedia %}{% admin\_media\_prefix %}css/dashboard.css{% endblock %}
{% block coltype %}colMS{% endblock %}
{% block bodyclass %}dashboard{% endblock %}
{% block breadcrumbs %}{% endblock %}
{% block content %}

{% if app\_list %}
{% for app in app\_list %}

{% blocktrans with app.name as name %}{{ name }}{% endblocktrans %}

{% for model in app.models %}|{% if model.perms.change %} {{ model.name }} |{% else %} {{ model.name }} |{% endif %}
{% if model.perms.add %} {% trans 'Add' %} |{% else %}  |{% endif %}
{% if model.perms.change %} {% trans 'Change' %} |{% else %}  |{% endif %}
{% endfor %}

{% endfor %}
{% else %}

{% trans "You don't have permission to edit anything." %}

{% endif %}

{% endblock %}
{% block sidebar %}

## {% trans 'Recent Actions' %}

### {% trans 'My Actions' %}

{% load log %}
{% get\_admin\_log 10 as admin\_log for\_user user %}
{% if not admin\_log %}

{% trans 'None available' %}

{% else %}

{% for entry in admin\_log %}- {% if not entry.is\_deletion %}{% endif %}{{ entry.object\_repr|escape }}{% if not entry.is\_deletion %}{% endif %}  
  {% filter capfirst %}{% trans entry.content\_type.name %}{% endfilter %}
{% endfor %}
{% endif %}

{% endblock %}
