# Supplementary figures and images for: A-MADMAN: Annotation-based microarray data meta-analysis tool
Source: BMC Bioinformatics. 2009 Jun 29;10:201. doi: 10.1186/1471-2105-10-201 (PMC2711946; doi:10.1186/1471-2105-10-201)

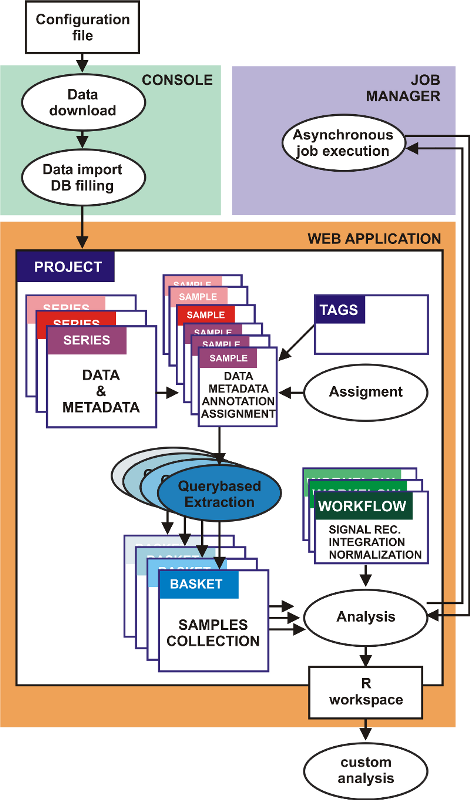

Supplement: Additional file 1 — A-MADMAN 1.4 source code. Version 1.4 of A-MADMAN source code. [file 1471-2105-10-201-S1.zip › amadman/ua_manager/media/flowchart.png]

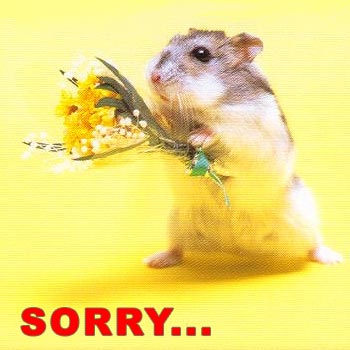

Supplement: Additional file 1 — A-MADMAN 1.4 source code. Version 1.4 of A-MADMAN source code. [file 1471-2105-10-201-S1.zip › amadman/ua_manager/media/sorry.jpg]

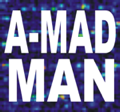

Supplement: Additional file 1 — A-MADMAN 1.4 source code. Version 1.4 of A-MADMAN source code. [file 1471-2105-10-201-S1.zip › amadman/ua_manager/media/amadmanlogo.png]

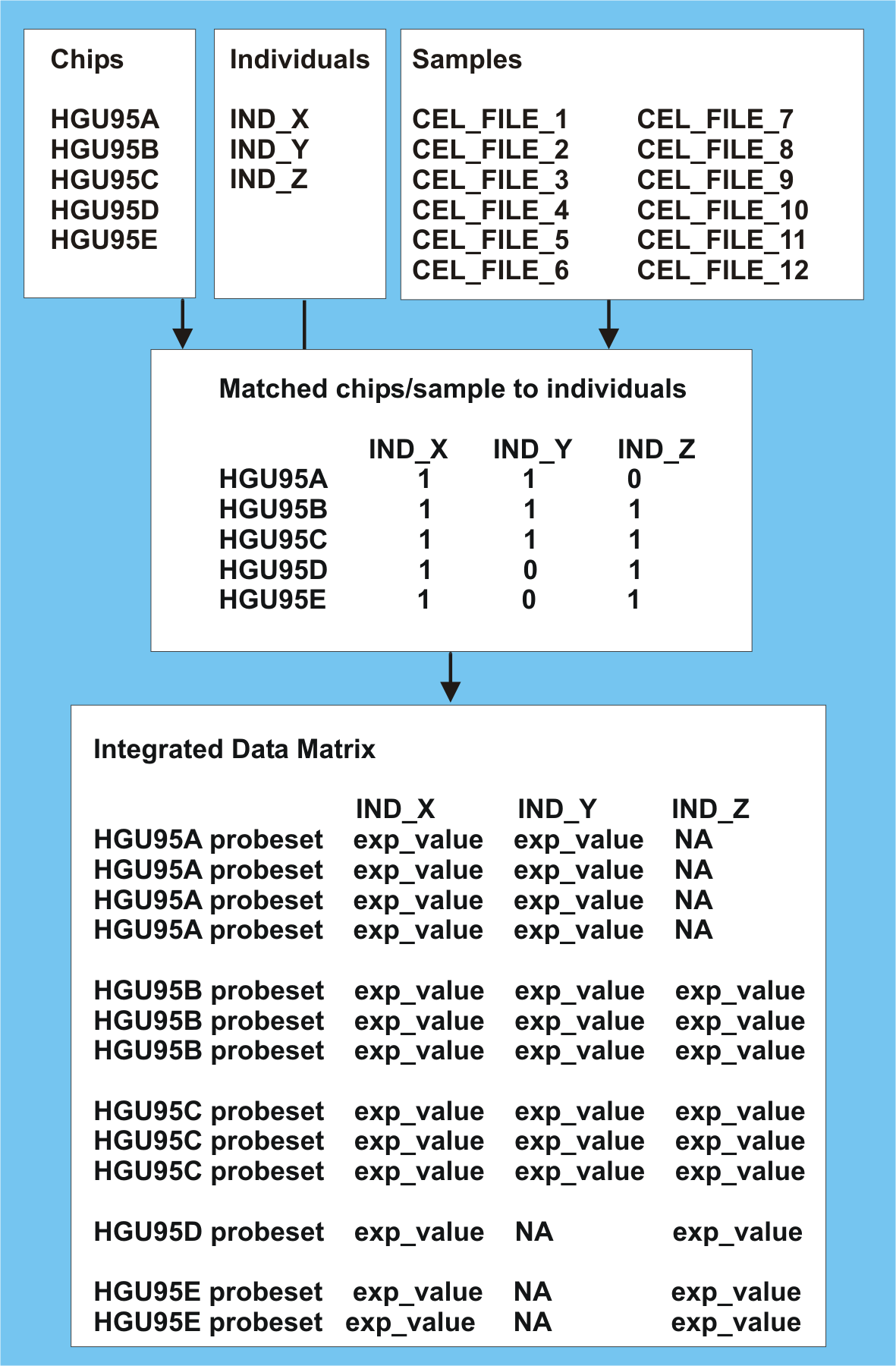

Supplement: Additional file 1 — A-MADMAN 1.4 source code. Version 1.4 of A-MADMAN source code. [file 1471-2105-10-201-S1.zip › amadman/ua_manager/media/docs/assign.png]

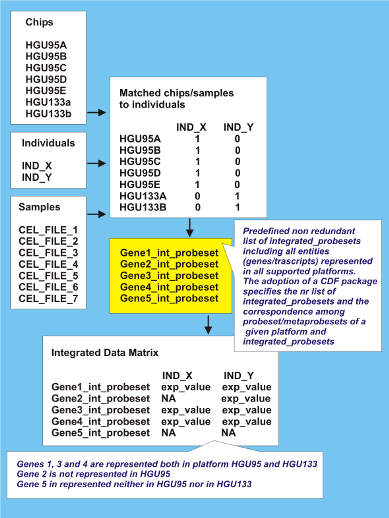

Supplement: Additional file 1 — A-MADMAN 1.4 source code. Version 1.4 of A-MADMAN source code. [file 1471-2105-10-201-S1.zip › amadman/ua_manager/media/docs/platform-integration-thumb.png]

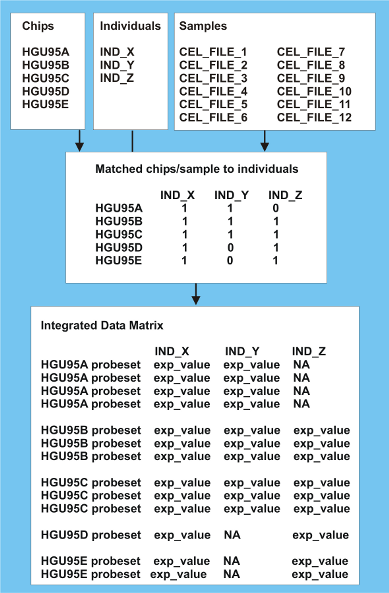

Supplement: Additional file 1 — A-MADMAN 1.4 source code. Version 1.4 of A-MADMAN source code. [file 1471-2105-10-201-S1.zip › amadman/ua_manager/media/docs/assign-thumb.png]

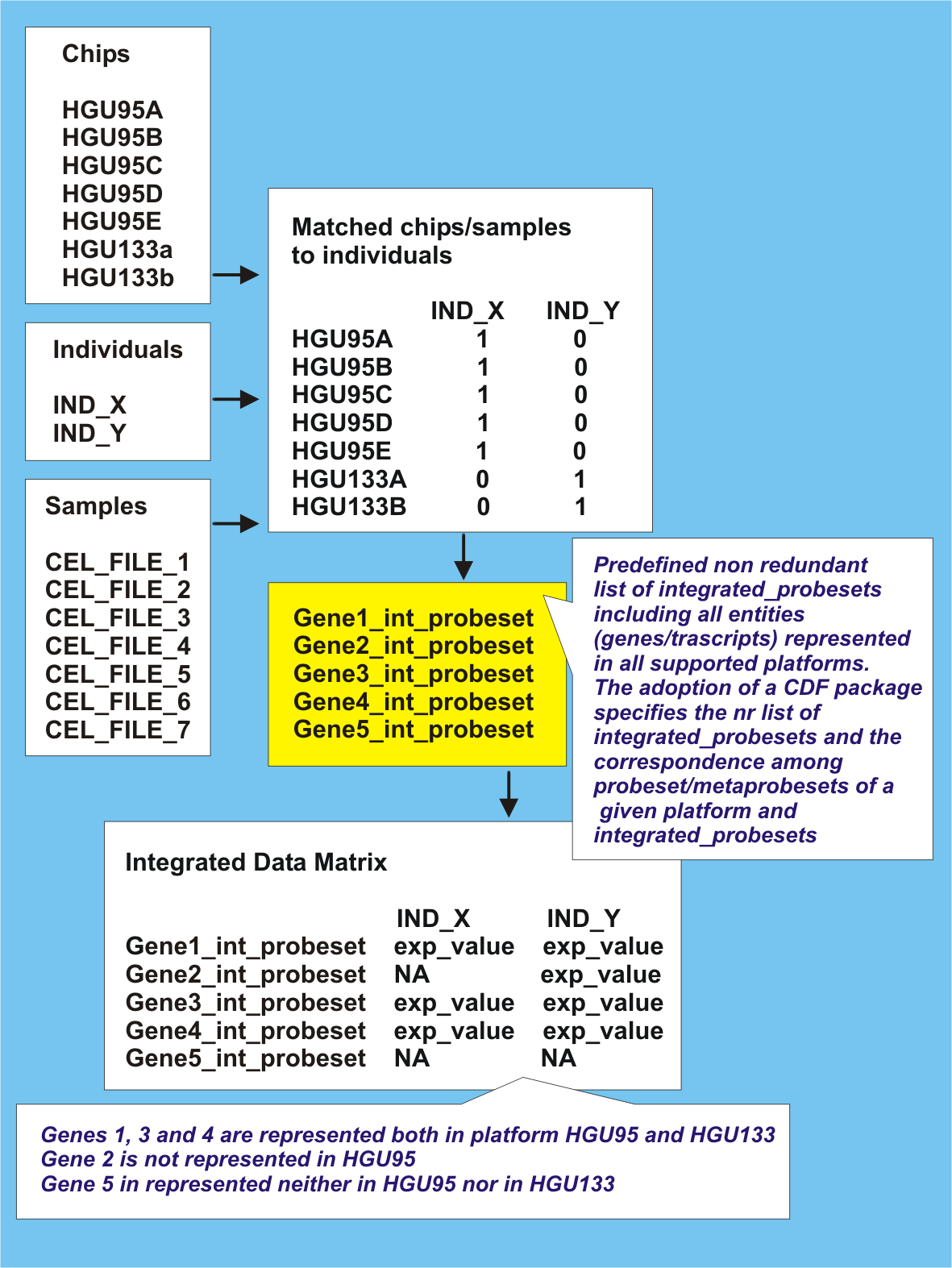

Supplement: Additional file 1 — A-MADMAN 1.4 source code. Version 1.4 of A-MADMAN source code. [file 1471-2105-10-201-S1.zip › amadman/ua_manager/media/docs/platform-integration.png]

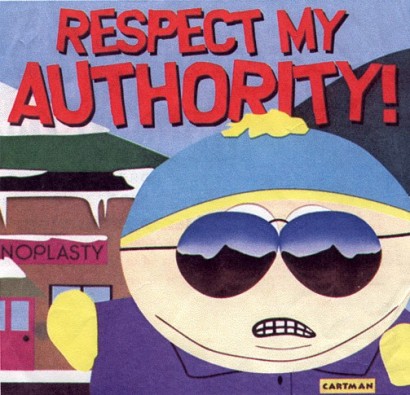

Supplement: Additional file 1 — A-MADMAN 1.4 source code. Version 1.4 of A-MADMAN source code. [file 1471-2105-10-201-S1.zip › amadman/ua_manager/media/cartman.jpg]

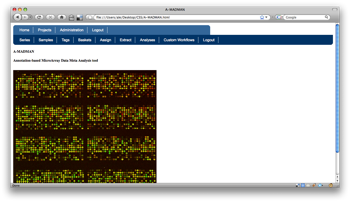

Supplement: Additional file 1 — A-MADMAN 1.4 source code. Version 1.4 of A-MADMAN source code. [file 1471-2105-10-201-S1.zip › amadman/ua_manager/media/screenshot2.png]

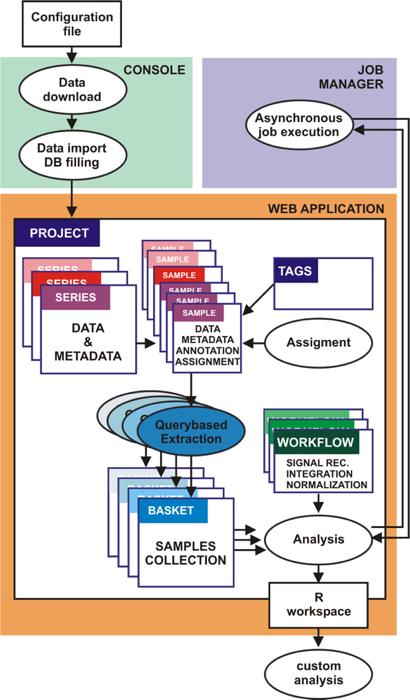

Supplement: Additional file 1 — A-MADMAN 1.4 source code. Version 1.4 of A-MADMAN source code. [file 1471-2105-10-201-S1.zip › amadman/ua_manager/media/schema.png]

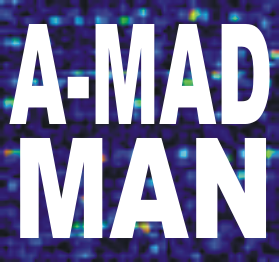

Supplement: Additional file 1 — A-MADMAN 1.4 source code. Version 1.4 of A-MADMAN source code. [file 1471-2105-10-201-S1.zip › amadman/ua_manager/media/amadman.png]

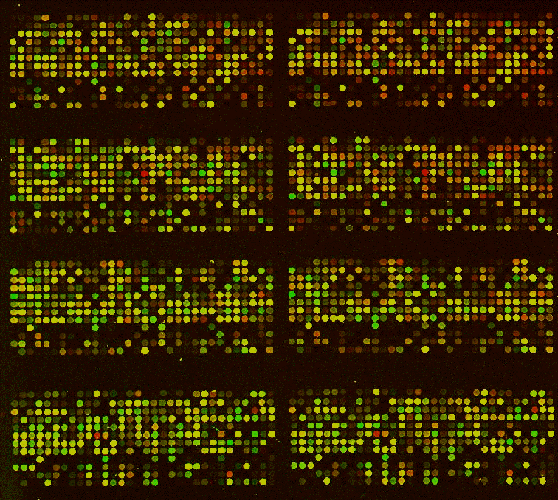

Supplement: Additional file 1 — A-MADMAN 1.4 source code. Version 1.4 of A-MADMAN source code. [file 1471-2105-10-201-S1.zip › amadman/ua_manager/media/microarray.gif]

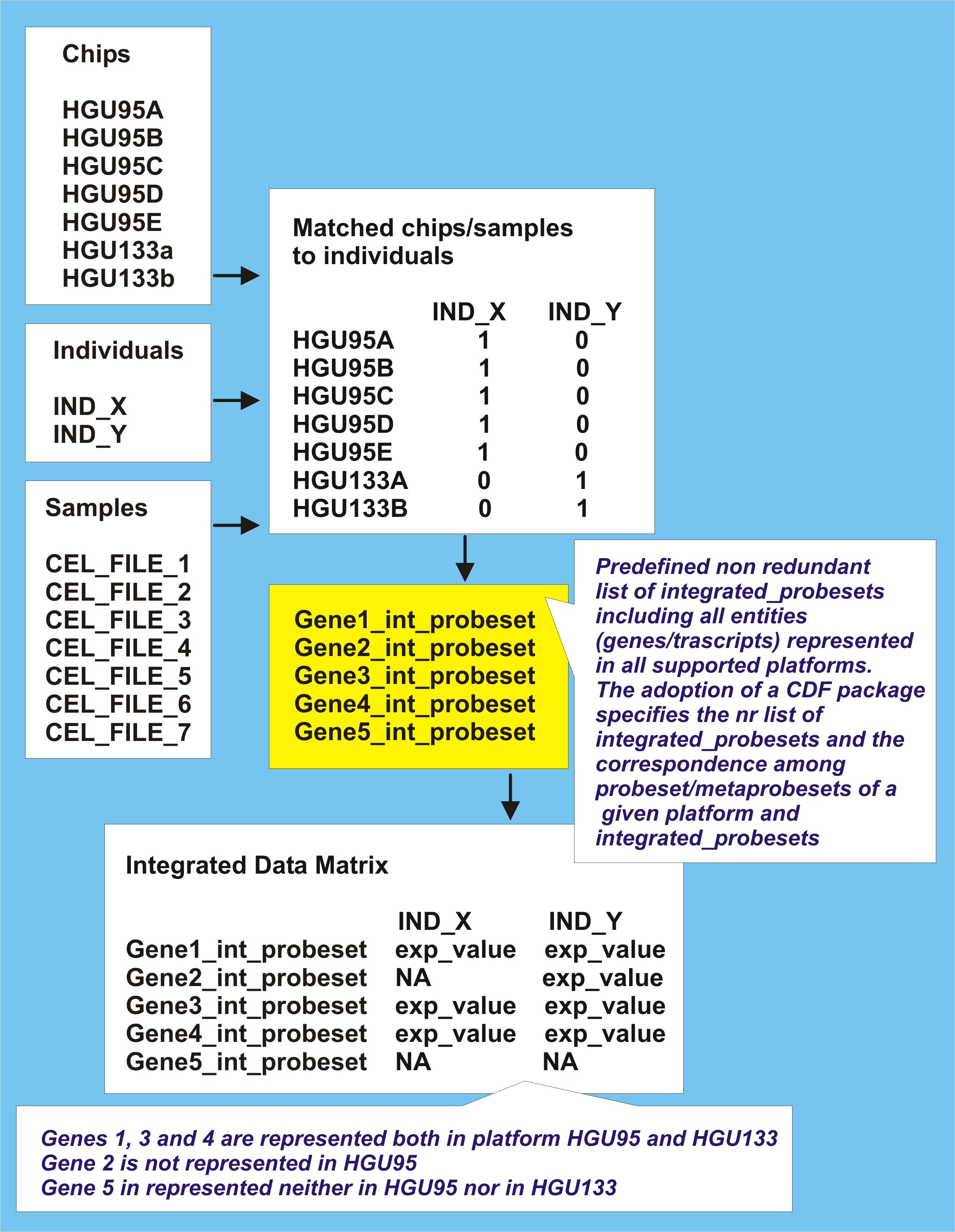

Supplement: Additional file 2 — Supplementary Figure 1. The role of raw data-to-individuals assignment in the process of platforms integration. [file 1471-2105-10-201-S2.jpeg]
